# Left breast irradiation with tangential intensity modulated radiotherapy (t-IMRT) versus tangential volumetric modulated arc therapy (t-VMAT): trade-offs between secondary cancer induction risk and optimal target coverage

**DOI:** 10.1186/s13014-019-1363-4

**Published:** 2019-09-02

**Authors:** Daniel Karpf, Mazen Sakka, Martin Metzger, Gerhard G. Grabenbauer

**Affiliations:** 1 Department of Radiation Oncology, Coburg Cancer Center, Coburg, Germany; 2 Medical Faculty of the Friedrich-Alexander-University of Erlangen-Nuremberg, Erlangen, Germany; 3 Division of Radiation Physics, Department of Radiation Oncology, Coburg Cancer Center, Coburg, Germany

**Keywords:** Breast cancer, Deep inspiration breath hold, Cardiac dose, Tangential intensity modulated radiotherapy (t-IMRT), Tangential volumetric modulated arc therapy (t-VMAT). Normal tissue integral dose (NTID), Homogeneity index (HI) and conformity index (CI)

## Abstract

**Background:**

Adjuvant radiotherapy is the standard treatment after breast-conserving surgery. According to meta-analyses, adjuvant 3d-conventional irradiation reduces the risk of local recurrence and thereby improves long-term survival by 5–10%. However, there is an unintended exposure of organs such as the heart, lungs and contralateral breast. Irradiation of the left breast has been related to long-term effects like increased rates of coronary events as well as second cancer induction. Modern radiotherapy techniques such as tangential intensity modulated radiotherapy (t-IMRT) and tangential volumetric modulated arc therapy (t-VMAT) and particularly deep inspiration breath hold (DIBH) technique have been developed in order to improve coverage of target volume and to reduce dose to normal tissue. The aim of this study was to compare t-IMRT-plans with t-VMAT-plans in DIBH position for left-sided breast irradiation in terms of normal tissue exposure, i.e. of lungs, heart, left anterior descending coronary artery (LADCA), as well as homogeneity (HI) and conformity index (CI) and excess absolute risk (EAR) for second cancer induction for organs at risk (OAR) after irradiation.

**Methods:**

Twenty patients, diagnosed with left-sided breast cancer and treated with breast-preserving surgery, were included in this planning study. For each patient DIBH-t-IMRT plan using 5 to 7 beams and t-VMAT plan using four rotations were generated to achieve 95% dose coverage to 95% of the volume. Data were evaluated on the basis of dose-volume histograms: Cardiac dose and LADCA (mean and maximum dose, D25% and D45%), dose to ipsilateral and contralateral lung (mean, D20%, D30%), dose to contralateral breast (mean dose), total monitor units, V5% of total body and normal tissue integral dose (NTID). In addition, homogeneity index and conformity index, as well as the excess absolute risk (EAR) to estimate the risk of second malignancy were calculated.

**Results:**

T-IMRT showed a significant reduction in mean cardiac dose of 26% (*p* = 0.002) compared to t-VMAT, as well as a significant reduction in the mean dose to LADCA of 20% (*p* = 0.03). Following t-IMRT, mean dose to the left lung was increased by 5% (*p* = 0.006), whereas no significant difference was found in the mean dose to the right lung and contralateral breast between the two procedures. Monitor units were 31% (*p* = 0.000004) lower for t-IMRT than for t-VMAT. T-IMRT technique significantly reduced normal tissue integral dose (NTID) by 19% (*p* = 0.000005) and the V5% of total body by 24% (*p* = 0.0007). In contrast, t-VMAT improved CI and HI by 2% (*p* = 0.001) and 0.4% (*p* = 0.00001), respectively. EAR with t-IMRT was significantly lower, especially for contralateral lung and contralateral breast (2–5/10,000 person years) but not for ipsilateral lung.

**Conclusion:**

Compared to t-VMAT, t-IMRT in left-sided breast irradiation significantly reduced dose to organs at risk as well as normal tissue integral dose, and V5% total body. EAR with t-IMRT was significantly lower for contralateral lung and contralateral breast. T-VMAT, however, achieved better homogeneity and conformity. This may be relevant in individual cases where sufficient coverage of medial lymphatic target volumes is warranted.

## Introduction

Breast cancer is the most common malignancy in the Western World [1]. According to Robert Koch Institute incidence and mortality rate in Germany are reported as being in the range of 114.6/100,000 (incidence) and 23/100,000 (mortality) [2]. Breast cancer as a systemic disease requires a multimodal treatment concept consisting of surgery, chemotherapy and radiotherapy. If necessary, these can be supplemented by hormone and antibody therapy. In case of early breast cancer, breast conserving surgery followed by adjuvant radiotherapy is regarded as standard treatment method. Numerous randomized data including meta-analyses have shown a lower recurrence rate and better long-term survival of 5–10% after adjuvant 3d-conventional irradiation (3d-CRT) in breast cancer patients [3–5]. However, apart from beneficial effects, irradiation may cause detrimental side effects on normal tissue. Specifically, when irradiating left-sided breast cancer, not only the lungs, and contralateral breast but also the heart with coronary vessels may receive significant dose of ionizing radiation. Recent data from the literature indicated that cardiac dose was associated with an increased rate of coronary events and cardiac mortality [6–8]. To minimize dose to organs at risk as low as possible, new techniques have been developed which attempt to achieve this aim by increasing homogeneity and conformity of the irradiation and better saving the risk organs [9–12]. These include the improvement of imaging procedures and further development of treatment planning systems. In addition, new irradiation methods have been introduced, for example deep-inspiration breath-hold (DIBH) in combination with or without tangential intensity modulated radiotherapy (t-IMRT) and tangential volumetric modulated arc therapy (t-VMAT).

The advantage of t-IMRT and t-VMAT technique, compared to former irradiation techniques (3d-CRT), is higher dose conformity and a more uniform dose distribution inside the target volume, especially with atypically shaped target volumes and thus a better protection of normal tissue and organs at risk [13–15]. In contrast to t-IMRT, in which the irradiation device is positioned at a certain angle around the patient, t-VMAT technology rotates the irradiation device once or several times continuously around the patient. With the t-VMAT technique, it is possible to simultaneously regulate the gantry speed, the treatment field shape using the movement of the MLC leaves and the dose rate during irradiation. Compared to 3D-CRT, additionally to the high conformal dose distribution, improved target volume coverage, t-VMAT can reduce the individual treatment time [16–18]. One point of concern on t-VMAT and t-IMRT is that at the expense of improved protection of the normal tissue from high radiation doses, the volume of normal tissue receiving low doses increase [19, 20]. This simultaneously increases the risk of developing a second malignancy. In contrast to deterministic side effects, there is no threshold dose value for the development of irradiation-induced malignancies, so that even low doses are significant. This is due to the fact that in the low dose range the changes in the cell cycle are in the foreground compared to cell destruction. Taking into account the excellent outcome of patients with early breast cancer, the risk of irradiation-induced cancer becomes particularly relevant. There are several mathematical formulas for assessing the risk of a second malignancy. One possibility is to estimate the excess absolute risk (EAR) per 10,000 person-years. EAR describes the absolute risk of second cancer of a person exposed to irradiation compared to a control group for specific organs based on the calculation of the organ equivalent dose (OED), which describes the dose-response relationship for cancer caused by irradiation and depends on age at the time of irradiation and age attained [21, 22].

Most of the previous studies compared between 3d-CRT and mainly discussed only the benefits of DIBH in terms of reduction of cardiac dose during left sided breast radiotherapy. Novelty of the present study is that it goes ahead in a further step to compare between two different modern radiation techniques, namely t-IMRT and t-VMAT in DIBH-position, and simultaneously assessed not only the dose reduction to organs at risk but also other parameters such as normal tissue integral dose (NTID), homogeneity- and conformity index as well as the risk of second malignancy.

At low doses (< 2Gy), the dose-response relationship is linear, based on data from atomic bomb attack survivors. At higher doses, as used in radiotherapy, the relationship takes on different forms because there is a different weighting of cell destruction, cell repair and cell regeneration. The dose-response relationship is no longer linear, but decreasing and assumes a plateau. The models for higher doses are based on data from a group of patients with Hodgkin’s lymphoma who received external radiotherapy. Using these data, Schneider and Walsh developed a mathematical model, taking into account various organ-specific and dose-dependent parameters, which allows an exact estimation of the EAR for both low and higher doses [23–26].The aim of the study was to compare t-IMRT-plans with t-VMAT-plans in deep inspiration breath-hold position (DIBH) for left sided breast cancer after breast-conserving surgery in terms of normal tissue exposure as well as homogeneity and conformity. In addition, the EARs for left and right lung and contralateral breast were calculated and compared for both techniques.

## Materials and methods

### Patients and inclusion criteria

Twenty consecutive patients, diagnosed with left sided invasive, lymph-node negative breast cancer or ductal carcinoma in situ (DCIS) and treated with breast-conserving surgery, were included in this retrospective planning study. Inclusion criteria were an age of less than 70 years, a good understanding of the procedure and the possibility of reproducing respiratory arrest, a good performance status, as well as clinically and pathologically negative lymph nodes without indication for regional lymph node irradiation,. All patients underwent CT simulation and irradiation in DIBH.

### CT simulation, delineation, and treatment planning

The CT simulation was performed in supine position with both arms positioned above the head and a copper wire placed around the breast tissue. Spiral CT scans were taken from the upper border of the hyoid bone to the diaphragm and reconstructed with a slice thickness of 3 mm. The clinical target volume (breast only), as well as all organs at risk, such as heart, LADCA, right lung, left lung and contralateral breast were contoured according to the Radiation Therapy Oncology Group (RTOG) and Danish Breast Cancer Cooperative Group (DBCG) delineation guidelines for adjuvant radiotherapy of early breast cancer [27, 28]. Heart and LADCA were countoured according to Feng et al [29]. For each patient two plans were generated.

T-IMRT plans used 5 to 7 beams and were created with the Pinnacle planning system (Version 9; Philips Medical Systems, Palo Alto, CA) and processed into the eclipse planning system (Version 13.6, Varian Medical Systems, Palo Alto, CA).

IMRT technique used was step and shoot IMRT. In clinical routine we mainly used the Eclipse-TPS where all contouring and final dose calculation was being performed. Pinnacle was exclusively used for calculation of step and shoot t-IMRT in patients with breast carcinoma since we felt that sparing of the contralateral breast and heart may be much superior with this TPS. Following completion of the Pinnacle plan, this plan was then exported to Eclipse where the dose distribution was recalculated. This procedures enabled to compare t-VMAT- and t-IMRT-plans in one DVH and to consequently choose the plan that appears more acceptable from a clinical perspective.

**Fig. 1:**
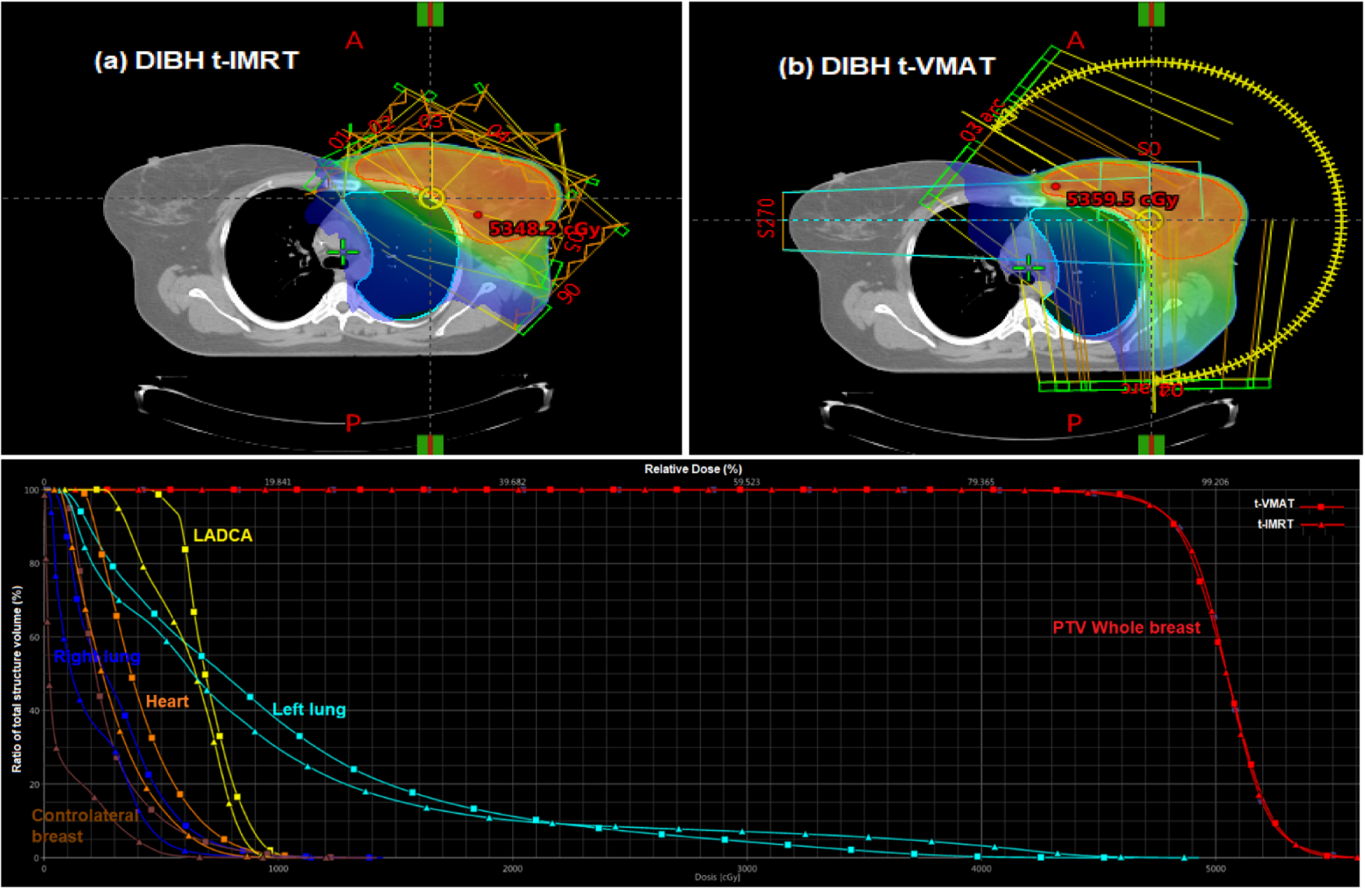
Beam arrangement, isodose distribution and DVH of target volume and organs at risk in both **a** DIBH t-IMRT and **b** DIBH t-VMAT plans for a left-sided breast cancer case

The plans for t-VMAT, which used 4 semi arcs, were developed completely in the above mentioned Eclipse system. Both, t-VMAT and t-IMRT, were set to 6 MV photon beams and used a total dose of 50.4 Gy, fractionated in a daily dose of 1.8 Gy, to ensure good comparability between the two methods (see Fig. 1). T-IMRT was typically executed using 6–8 beam equally weighted angles between 300° and 175°. During optimization procedure, however, tangential beams may adopt a larger contribution to the total dose. The same applied in principle for t-VMAT, where rotations between 300° and 179° with different collimator-angles (e.g. 10°, 350°, 80°, 100° using four arcs) were usually contemplated.

Plans were optimized to achieve 95% coverage of 95% of the target volume. For both techniques inversed planning optimization was used. There were no specific reference constraints for all cases and all plans, however for the optimization procedures, different templates that automatically generated constraints for organs at risk were used followed by individual adjustments based on planning expertise and experience.

### Dosimetric assessment

The main parameters used to compare the t-VMAT and t-IMRT techniques were the V5% total body volume, total number of monitor units (MU), normal tissue integral dose (NTID), as well as the homogeneity index (HI), conformity index (CI) and the dose to organs at risk (OAR). In addition, the EAR was estimated for comparison between the two methods. The results were determined and calculated on the basis of dose-volume histograms in DIBH technique. For all parameters, the mean value and standard deviation were calculated from individual values of the 20 patients. The statistical analysis was performed with the Wilcoxon signed rank test.

### Normal tissue integral dose, V5% of Total body, monitor units

#### Monitor units

The monitor units correlated with the treatment time, or more precisely the beam on time. For each patient, the sum of the monitor units from the individual t-VMAT rotations and the sum of the individual beams in the t-IMRT were taken into account. From the values of the 20 patients, an average value was calculated for both procedures.

#### Normal tissue integral dose (NTID)

The NTID was calculated from the difference between the dose to total body and the planning target volume and had the unit cGy per cm^3^. It gives information about how much irradiation the normal tissue receives on average per cm^3^.

#### V5% of Total body

V5% of total body was defined as the volume of the body that received 5% of the total dose.

### Homogeneity index and conformity index

#### Conformity index (CI)

The following formula was used for calculation:

1 $$ CI=\frac{V95\%}{V(PTV)} $$

V95% represents the volume receiving the prescribed dose and V (PTV) is the planning target volume.

#### Homogeneity index (HI)

Homogeneity index is a tool for dosimetric analysis of treatment plans to describe the uniformity of dose distribution in the target volume and thus a good indicator for plan quality. The higher the homogeneity, the lower the dose peaks are in order to obtain a sufficient amount of radiation dose throughout the target volume. There are various formulae for its calculation [30]. In this study the following version to calculate homogeneity index was used:

2 $$ HI=\frac{D5\%}{D95\%} $$

D5%: Minimum dose in 5% of the PTV indicating the maximum dose.

D95%: Minimum dose in 95% of the PTV indicating the minimum dose.

The formulas for HI and CI were chosen in such a way that the optimal value is 1.

### Excess absolute risk

EAR can be calculated using the organ equivalent dose (OED) based on data of dose-volume-histograms. This is the uniform dose in Gray distributed over an organ, which induces a second malignancy with the same probability of irradiation as in an organ with non-uniform dose [21].

3 $$ OED=\frac{1}{Vt}\sum V(Di) RED(Di) $$

*Vt* describes the total volume of the organ to be analyzed. *V (Di*) is the volume of an organ that receives a certain irradiation dose *Di. RED (Di)* describes the dose-response relationship based on the data of the atomic bomb survivors and patients with Hodgkin’s lymphoma being irradiated.

For the most accurate estimation of the EAR, the full mathematical model is appropriate, which integrates and takes into account both cell destruction effects as well as cell repair and cell regeneration effects. In addition, dose fractionation is also taken into account [22, 25].

4 $$ RED(Di)=\frac{e^{-\alpha \acute{\mkern6mu} Di}}{\alpha \acute{\mkern6mu}R}\left[1-2R+{R}^{2{e}^{\alpha \acute{\mkern6mu} Di}}-\left(1-R\right)2{e}^{-\frac{\alpha \acute{\mkern6mu}R}{1-R} Di}\right] $$

R is a repopulation parameter which ranges between 0 and 1 (R = 0: There is no repopulation of tissue cells between two fractions; R = 1: There is full repopulation of tissue cells). *α*´ represents cell destruction effects and is calculated as follows:

5 $$ \alpha \acute{\mkern6mu}=\alpha +\beta d=\alpha +\beta \frac{D}{N} $$

where *d* is the dose per fraction, *D* is total dose and *N* is the number of fractions *α* and *β* are parameters of the linear-quadratic model.

In order to obtain the EAR, age at irradiation and age reached and a factor μ are taken into consideration in addition to the OED.

6 $$ EAR=\mu \frac{1}{Vt}\sum V(Di) RED(Di) $$

where μ is the slope of dose-response relationship for the cancer-induction. The age distribution is considered with the following formula:

7 $$ \mu \left( agex, agea\right)=\mu \acute{\mkern6mu}{e}^{\left[\gamma e\left( agex-30\right)+ ya\ln \left(\frac{agea}{70}\right)\right]} $$

where ye und ya are age modifying parameters, the age attained has been set at 70 years for all patients.

The parameters were chosen, as summarized by Schneider et al. [22], and the values for the variables u, R and alpha, based on the study by Fogliata et al. [31]. Table 1 gives an overview of the variables used.

**Table 1:** Parameters used for calculation of Excess Absolute Risk (EAR)

	α in $\frac{1}{Gy}$	α/β	μ	R	d in *Gy*	ye	ya
Lung	0.022	3	3.8	0.83	1.8	0.002	4.23
Breast	0.067	3	5	0.62	1.8	−0.037	1.70

## Results

### Organs at risk

Table 2 shows the mean heart dose for all patients in both irradiation techniques, t-IMRT and t-VMAT. As compared to t-VMAT, t-IMRT showed a significant reduction in mean cardiac dose of 26% (*p* = 0.002), as well as a significant reduction in the mean dose to LADCA of 20% (*p* = 0.03). Following t-IMRT, mean dose to the left lung was increased by 5% (*p* = 0.006), whereas no significant difference was found in the mean dose to the right lung and contralateral breast between the two procedures. Table 2 gives an overview on the actual dose parameters.

**Table 2:** Dose parameters for organs at risk, i.e. average dose (in Gy) to heart, LADCA, and left lung for 20 patients with left-sided breast cancer comparing t-IMRT and t-VMAT, both in DIBH-technique

Heart dose	DIBH-t-IMRT	DIBH-t-VMAT	Difference	*p*-value
Dmean	2.96 ± 0.61	4.03 ± 0.74	- 1.07 (− 26%)	0.002
D25%	3.84 ± 0.69	4.81 ± 0.81	- 0.97 (−20%)	0.002
D45%	2.68 ± 0.69	3.81 0.63	- 1.13 (−29%)	0.003
LADCA dose				
Dmax	12.28 ± 3.49	15.45 ± 5.6	- 3.17 (−20%)	0.003
Dmean	5.80 ± 0.68	7.31 ± 0.97	- 1.51 (−20%)	0.03
D25%	6.58 ± 0.68	8.48 ± 1.18	−1.9 (−22%)	0.01
Left Lung				
Dmean	10.42 ± 0.71	9.89 ± 1.03	+ 0,53 (+ 5%)	0.006

### Monitor units, Normal tissue integral dose, V5% of Total body

**Fig. 2:**
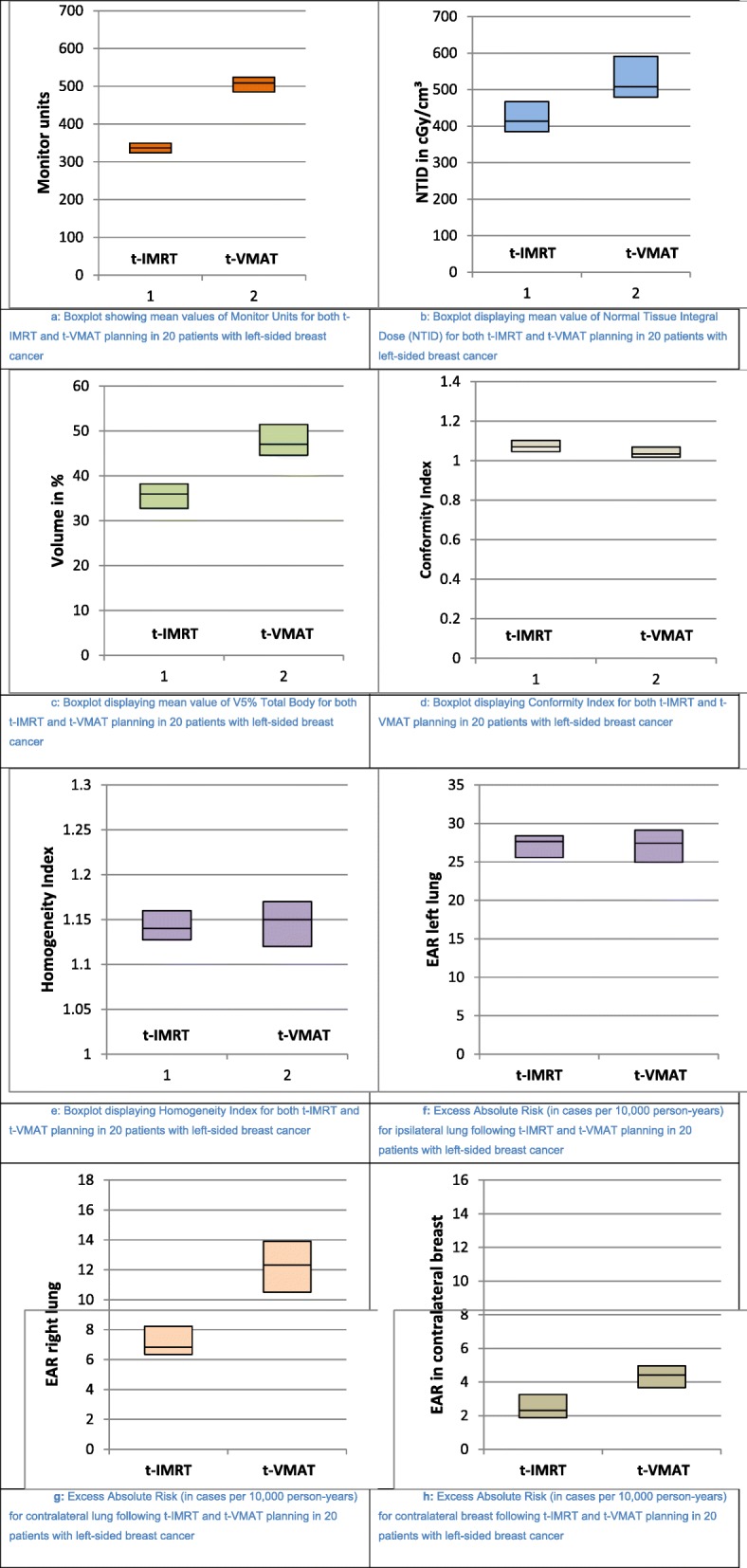
**a** Boxplot showing mean values of Monitor Units for both t-IMRT and t-VMAT planning in 20 patients with left-sided breast cancer. **b** Boxplot displaying mean value of Normal Tissue Integral Dose (NTID) for both t-IMRT and t-VMAT planning in 20 patients with left-sided breast cancer. **c** Boxplot displaying mean value of V5% Total Body for both t-IMRT and t-VMAT planning in 20 patients with left-sided breast cancer. **d** Boxplot displaying Conformity Index for both t-IMRT and t-VMAT planning in 20 patients with left-sided breast cancer. **e** Boxplot displaying Homogeneity Index for both t-IMRT and t-VMAT planning in 20 patients with left-sided breast cancer. **f** Excess Absolute Risk (in cases per 10,000 person-years) for ipsilateral lung following t-IMRT and t-VMAT planning in 20 patients with left-sided breast cancer. **g** Excess Absolute Risk (in cases per 10,000 person-years) for contralateral lung following t-IMRT and t-VMAT planning in 20 patients with left-sided breast cancer. **h** Excess Absolute Risk (in cases per 10,000 person-years) for contralateral breast following t-IMRT and t-VMAT planning in 20 patients with left-sided breast cancer

T-IMRT showed a significant reduction of V5% total body by 24% (*p* = 0.0007), as well as a significant reduction of NTID by 19% (*p* = 0.0000005). This was associated with better organ sparing effects in the low dose range. In addition the monitor units were 31% (*p* = 0.0000004) lower for t-IMRT than for t-VMAT, which resulted in a shortened treatment time. All three parameters showed advantages for t-IMRT compared to t-VMAT. The results were reflected in each individual patient for each parameter. Table 3 summarizes the results of the evaluation of MUs, NTID and V5% total body parameters of both techniques. Figure 2a-c show the results graphically in box plots.

**Table 3:** Comparison of Monitor Units, Normal Tissue Integral Dose, and V5% of Total Body between t-IMRT and t-VMAT for 20 patients with left-sided breast cancer

	t-IMRT	t-VMAT	Difference	*p*-value
V5% total body (%)	36.02 ± 4.14	47.275 ± 5.89	−11.255 (−24%)	0.0007
NTID (cGy/cm^3^)	421.3 ± 52.07	523.26 ± 71.99	−102 (− 19%)	0.0000005
Monitor Units	348.155 ± 43.33	504.19 ± 35,39	−156.035 (−31%)	0.0000004

### Conformity index and homogeneity index

In contrast to the parameters considered above, t-VMAT improved the homogeneity and conformity index in comparison to t-IMRT by 0.4% (*p* = 0.00001) and 2% (*p* = 0.001). In the patients considered individually, the results were inconsistent, so that the advantages shown on average, could not be projected to each individual patient. Table 4 and Fig. 2d-e show the results for homogeneity index and conformity index of t-IMRT and t-VMAT-planning.

**Table 4:** Mean values for conformity index and homogeneity index for both t-IMRT and t-VMAT planning in 20 patients with left-sided breast cancer

	t-IMRT	t-VMAT	Difference	*p*-value
Conformity index	1.072 ± 0.015	1.0456 ± 0.015	+ 0.027 (+ 2%)	0.001
Homogeneity index	1.1545 ± 0.01	1.15 ± 0.02	+ 0.005 (+ 0.4%)	0.00001

### Excess absolute risk

For the contralateral lung and contralateral breast, t-IMRT showed lower EAR values in all 20 patients. On average t-IMRT reduced EAR by 42% (*p* = 0.04) for contralateral lung and by 40% (*p* = 0.00002) for contralateral breast. The absolute difference in EAR between t-IMRT and t-VMAT in contralateral organs was 2–5 cases per 10,000 person years. In contrast, the absolute difference in the ipsilateral lung was less than 1 case per 10,000 person years. Statistically, a significant reduction by 0.8% (*p* = 0.0004) in EAR was found following t-IMRT. However, 10 patients (50%) showed a lower EAR of the ipsilateral lung using the t-VMAT technique. Table 5 and Fig. 2f-h show the data for EAR in three organs where induction of secondary cancer is mainly to be expected.

**Table 5:** Excess Absolute Risk for ipsilateral lung, contralateral lung and contralateral breast for both t-IMRT and t-VMAT planning in 20 patients with left-sided breast cancer

	t-IMRT	t-VMAT	Difference	p-value
EAR (ipsilateral lung)	27.072 ± 2.187	27.295 ± 2.473	−0.223 (−0.8%)	0.0004
EAR (contralateral lung)	7.126 ± 1.105	12.312 ± 2.02	−5.186 (−42%)	0.04
EAR (contralateral breast)	2.985 ± 2.148	4.949 ± 2.373	−1.964 (−40%)	0.000002

#### Total treatment time

Irradiation was exclusively performed in DIBH-technique and the time a given patient was able to keep a deep inspiration status varied not only between patients but also from day to day. Therefore, total treatment time calculation just based on monitor units seemed not appropriate. Based on the “Record and Verify System” the *time span between start of first verification image and application of last monitor unit* was extracted on an individual basis. It turned out that IMRT-plans and VMAT-plans were executed in 13 and 11 patients, respectively (in three patients both techniques were used). For each patient total treatment times for all fraction were taken and a mean value for the individual treatment time of a given technique (IMRT vs VMAT) was calculated. Median and mean treatment times for IMRT vs VMAT were 6.62 min. and 7.05 min. (range, 5.11–10.08 min) vs. 6.14 min. and 6.38 min. (range, 5.36–8.75 min.), respectively. According to the Student’s T-test no significant difference was detected (*p* = 0.10).

## Discussion

In order to achieve optimal irradiation results for patients with left-sided breast cancer, i. e. best local control, lowest acute and late toxicity and lowest second cancer induction, modern irradiation techniques are used, including DIBH, t-IMRT and t-VMAT. Some studies have shown the superiority of 3D-DIBH over conventional 3D-CRT in regard to improving OAR constraints [9–11, 18, 32–34]. Regarding dose conformity and homogeneity there is a clear theoretical advantage for t-IMRT/ t-VMT -plan over 3D (32–33). However, the difference between t-IMRT and t-VMAT is less clear.

In a population of 20 patients with left-sided breast cancer, this planning study compared V5% of total body volume, monitor units (MU), normal tissue integral dose (NTID), as well as homogeneity index (HI), conformity index (CI) and dose to organs at risk for t-IMRT and t-VMAT plans. In addition, the EAR for cancer induction in several organs at risk was estimated for comparison between the two methods.

T-VMAT showed, compared to t-IMRT, a better dose homogeneity and dose conformity. CI and HI are regarded as important quality indicators for irradiation plans and provide recognized assistance in choosing between different irradiation plans. One problem is the inaccurate knowledge of the relationship between these theoretical parameters and the clinical effects of irradiation. In theory, better HI and CI would be expected to be associated with a lower local recurrence rate and fewer radiation-associated side effects. However, there are so far in the literature no studies that provide a clear clinical impact for these parameters. Another factor to consider is the existence of different formulas, both for HI and for CI. Kataria et al [30]. showed a sufficient degree of agreement between different formulae for homogeneity index, so that we limited ourselves to the calculation with one single formula. For both, HI and CI, we chose formulas, which have their optimum value of 1 to make a good comparison between the t-IMRT and t-VMAT.

High conformity and homogeneity, accompanied by a higher number of beams on the target volume, is often at the expense of an increased low-dose exposure for the tumor-surrounding tissue. This may result in a higher risk for the induction of second malignancies, a factor that raised major reluctance in using newer methods compared to conventional 3d-CRT [35]. Our study showed that t-IMRT reduced the V5% of total body, representing the low dose irradiation volume, as well as the normal tissue integral dose significantly, compared to t-VMAT.

Risk of second malignancy formation grows with increasing dose and irradiated volume. Given these facts, the full model for EAR gives a good assessment of the increased secondary cancer risk following irradiation of the respective organs [36, 37]. Mendes et al. demonstrated a relevant risk up to 2.2% of induced-cancer from left breast radiation in a dosimetric planning study, considering the whole thorax organs and Brazilian cancer-incidence [38]. The difference between t-VMAT and t-IMRT with respect to EAR can be observed particularly in the contralateral breast and contralateral lung, both receiving relatively low dose. The difference in exposure of the left lung between the two techniques was much smaller. Nevertheless, the absolute EAR was highest in left lung. Our results compare very favorably with data from the literature. Fogliata et al., as well as Sakthivel et al. showed a lower risk for second malignancy following t-IMRT as compared to t-VMAT [31, 39]. In our study, EAR was calculated exclusively using the full model described by Schneider et al., which best takes into account tissue and dose-dependent cell biological properties such as cell destruction and cell regeneration. Inaccuracies in the estimation could result from the inconsistency and variance in the variable parameters, which are not derived from data of a breast cancer population but from a population of patients surviving atomic bombs and patients irradiated for Hodgkin’s lymphoma. Another uncertainty factor is the heterogeneity in the age of the patients in our study, which has a large influence on the EAR.

It is also suggested that the risk of a second malignancy increases with a higher number of monitor units [37]. In our study, the number of MUs following t-IMRT was significantly lower compared to t-VMAT (31%, *p* = 0.0000004). Sakthivel et al. evaluated the relationship between number of MUs and EAR. According to their results, however, no strong relationship between the two parameters was seen [39].

Dumane et al. showed significant dosimetric gains with a lower dose to the heart, lungs and contralateral breast following DIBH irradiation of the left reconstructed chest wall and regional nodes [40]. Sakka et al. reported a significant additional decrease in heart and LADCA dose by t-IMRT in DIBH technique compared with t-VMAT [41]. In another dosimetric analysis of left breast irradiation in DIBH position Yu et al. found no difference between t-VMAT and t-IMRT [42].

In left-sided breast cancer irradiation, the most important organs at risk in terms of deterministic late effects are the heart, the coronary arteries as well as the ipsilateral lung. Given the well-known toxicity of numerous agents like doxorubicine and trastuzumab, long-term effects, especially coronary events, may be of crucial importance in the case of left-sided breast irradiation. Cardiovascular complications may progress over time and occur years after initial exposure. Various studies have shown that the risk for subsequent ischemic events increased linearly with the mean heart dose [7, 8, 43]. For older women, cardiovascular damage is a higher mortality risk than breast cancer itself [1]. In our study t-IMRT as compared to t-VMAT reduced the mean heart dose by 26% (*p* = 0.002) and the mean dose to LADCA by 20% (*p* = 0.03).

An example for an early late toxic effect is radiation-induced pneumonia, which is well correlated with the dose at the ipsilateral lung. The higher the mean lung dose when irradiating left-sided breast cancer, the higher the risk for developing radiation-induced pneumonia [44–46]. In contrast to the heart, in our study t-VMAT showed better sparing effects compared to t-IMRT and reduced the mean radiation dose to the ipsilateral lung by 5% (*p* = 0.006), which may be statistically different albeit not clinically relevant. Other planning studies comparing t-IMRT and t-VMAT have shown contradictory results. Ekambaram et al. demonstrated better homogeneity and conformity for the t-VMAT technique in left-sided breast cancer irradiation by using t-IMRT with 7 beams and t-VMAT with 2 semi arcs. However, in their study t-VMAT also showed better sparing effects, with a lower dose to heart and ipsilateral lung, as well as lower NTID and less MU. In line with these data, Popescu et al. reported superior results for t-VMAT in terms of sparing effects to OAR, while Zhao et al. and Sakka et al. demonstrated better sparing effects for the t-IMRT technique [41, 47–49].

However, comparisons between the different studies remain difficult because of differences in patient population, dose prescription, delineation and definition of target volumes and radiation techniques; e.g. number of fields and arcs used in t-IMRT and t-VMAT. This may explain the partially contradictory results with regard to HI, CI, MU and OAR sparing.

One interesting question remains: How much of the detected difference in dose distribution was due to the different irradiation techniques and how much was mainly a consequence of different optimization algorithms? This question must remain open since the individual performance, endurance and expertise may play a significant role in medical physics.

## Conclusion

Compared to t-VMAT, t-IMRT in left-sided breast irradiation significantly and considerably reduced dose to organs at risk as well as normal tissue integral dose, V5% total body and total number of monitor units. Excess absolute risk (EAR) for the induction of secondary tumours was significantly lower following t-IMRT than t-VMAT, especially in the contralateral lung and breast (2–5/10,000 person years) but not for ipsilateral lung. This risk appears small compared to the survival benefit achieved with adjuvant radiotherapy after breast conserving therapy in this group of early breast cancer patients. However, t-VMAT-plans achieved better homogeneity and conformity, based on index calculation. This may be relevant in individual cases where sufficient coverage of the parasternal nodes is warranted.

## Data Availability

Please share all primary data by contacting the first author (daniel_karpf@web.de).
